# Considerations and future challenges in managing ECMO for pediatric patients

**DOI:** 10.1007/s10047-026-01574-7

**Published:** 2026-07-30

**Authors:** Koichi Kashiwa, Kent Doi, Fumiaki Shikata, Minoru Ono

**Affiliations:** 1 Department of Clinical Engineering, The University of Tokyo Hospital, 7-3-1, Hongo, Bunkyo-Ku, Tokyo, Japan; 2 Department of Cardiovascular Surgery, The University of Tokyo Hospital, Tokyo, Japan

**Keywords:** Thrombus formation, Hemolysis, Centrifugal pump

## Abstract

In managing ECMO, preventing thrombus formation and hemolysis is crucial. While centrifugal pumps are increasingly used in pediatric ECMO, pumps designed for low flow range are not approved in Japan. Thus, centrifugal pumps for adults are used in pediatric ECMO cases. This increases the risk of thrombus formation in the enlarged flow paths before and after the centrifugal pump. To prevent thrombus formation in the ECMO circuit, it is necessary not only to appropriately optimize the dosage of anticoagulants but also to pay meticulous attention to circuit configuration. Furthermore, using centrifugal pumps at low flow rates increases shear stress on the blood, making hemolysis significantly more likely to occur in pediatric cases compared to adult cases. To reduce the incidence of hemolysis, it is necessary to use centrifugal pumps designed for low flow range. However, given the current situation where such centrifugal pumps are not approved in Japan, we must proceed with the understanding that hemolysis is more likely to occur in pediatric ECMO cases using centrifugal pumps. Moving forward, to reduce complications and improve outcomes in pediatric ECMO, the development of pediatric ECMO systems, including centrifugal pumps optimized for low-flow range, is required.

## Introduction

In pediatric extracorporeal membrane oxygenation (ECMO), roller pump and centrifugal pump are used, but it is presumed that many institutions now use centrifugal pump [1–3]. The PediMag™ Blood Pump (Abbott, Chicago, US), a centrifugal pump with 1/4-inch connector inner diameters for inflow and outflow ports capable of flow rates of up to 1.5 L/min, is used clinically worldwide but is not approved in Japan yet. Therefore, in Japan, centrifugal pumps with 3/8-inch connector inner diameter for the inflow and outflow ports, capable of flow rates of up to approximately 10 L/min, must be used even for pediatric patients. This necessitates enlarging the flow path before and after the centrifugal pump. Furthermore, operating centrifugal pumps at low flow rates increases shear stress on the blood. Consequently, pediatric ECMO using centrifugal pumps is significantly more prone to thrombus and hemolysis compared to adult cases, requiring meticulous management. This paper discusses key considerations and future challenges in managing pediatric ECMO from the perspective of the prevention of thrombus formation in the circuit and hemolysis.

## The prevention of thrombus formation

Reports indicate that thrombosis formation requiring partial or complete circuit replacement is associated with poor survival odds. Preventing thrombosis formation during ECMO is therefore critical in managing pediatric ECMO [3].

### Anticoagulation therapy

Reports from Europe and the US recommend the use of bivalirudin, a direct thrombin inhibitor [4, 5]. However, unfractionated heparin (UFH) remains the most widely used anticoagulant during ECMO. The continuous UFH dose is generally adjusted to maintain an activated clotting time (ACT) of approximately 180–200 s and an activated partial thromboplastin time (APTT) of approximately 1.5–2.5 times the baseline value [6]. However, anticoagulation with UFH is challenging in post-cardiac surgery ECMO patients due to the high risk of postoperative hemorrhage. Furthermore, pediatric ECMO with low pump flow rates commonly predisposes the circuit to thrombus, often necessitating frequent circuit exchange. Circuit exchanges result in the loss of coagulation factors and platelets, potentially increasing blood transfusion requirements and the incidence of associated complications. Furthermore, UFH exhibits non-specific bindings to endogenous plasma proteins, platelet factor 4, and von Willebrand factor multimers [7]. This limits its bioavailability and reduces its anticoagulant efficacy. In patients with ECMO, there is significant variation in the plasma concentrations of these proteins [8], and it has been reported that the protein binding rate is higher in smaller children [9], making it difficult to predict the response to UFH in children. Furthermore, the coagulation system is intricately linked to the inflammatory response. Excessive inflammation can trigger a cytokine storm, leading to the formation of neutrophil extracellular traps (NETs), elevated Factor VIII and von Willebrand factors, and complement activation that damages vascular endothelial cells. Additionally, the emergence of lupus anticoagulant activity can further accelerate blood coagulation and platelet aggregation [10]. Therefore, special attention is required in cases with increased inflammatory responses. Thus, in ECMO management, it is necessary to initiate anticoagulant therapy while considering various patient-specific coagulation and bleeding risks, and to adjust the dosage of anticoagulants while performing appropriate monitoring. Although many institutions appear to control the dosage of UFH while monitoring ACT and APTT, it is difficult to determine whether anticoagulation therapy is appropriate only based on ACT and APTT. It is necessary to comprehensively assess whether anticoagulation therapy is appropriate by combining various data, such as anti-Xa activity, platelet count, fibrinogen, antithrombin activity (AT), and blood viscoelasticity test data. Although anti-Xa activity has been reported to correlate with UFH dosage [11], only a limited number of hospitals in Japan routinely measure anti-Xa activity, making its monitoring difficult in ECMO management. At our institution, we have established a protocol that combines the aforementioned data with thrombin-antithrombin complex (TAT) to determine whether anticoagulant therapy is being administered appropriately. We consider TAT levels around 10 ng/mL to be within the normal range. If the value exceeds that level, we investigate the cause and consider increasing the anticoagulant dose as necessary. By monitoring these various data, we can determine whether excessive prolongation of ACT or APTT is due to excessive anticoagulant administration or coagulation factors depletion. If coagulation factors depletion is suspected as the cause of prolonged ACT or APTT, we administer plasma products to replenish coagulation factors while continuing anticoagulant therapy. Furthermore, protocols using only UFH may result in delayed initiation of anticoagulant therapy in ECMO cases following cardiac surgery or allow thrombus formation to progress during the UFH dosage adjustment phase. Therefore, our institution frequently combines UFH with continuous in-line infusion of nafamostat mesylate, a synthetic serine protease inhibitor. There is no high-quality evidence supporting the routine use of combination anticoagulation therapy with nafamostat mesylate and UFH. However, a report indicates that this combination, with nafamostat mesylate at 0.06 mg/kg/hr, suppressed circuit thrombosis in an adult COVID-19 patient undergoing V-V ECMO [12]. Our institution's dosage guideline for UFH and nafamostat mesylate are 100–400 IU/kg/day and 0.1–0.5 mg/kg/hr, respectively. We adjust these anticoagulant doses daily, monitoring various laboratory data mentioned above and the status of thrombus formation.

### Circuit

The ECMO circuit has multiple connection points between connectors and tubing. Even with appropriate anticoagulation therapy, thrombus can adhere to step-ups at connector joints, necessitating circuit replacement. Connector tip shapes vary by manufacturer, and some configurations create significant step-ups when connected to tubing, requiring careful attention. Some manufacturers are working to eliminate step-ups at connector joints, and we look forward to their future initiatives for these thrombosis-prone areas.

**Fig. 1:**
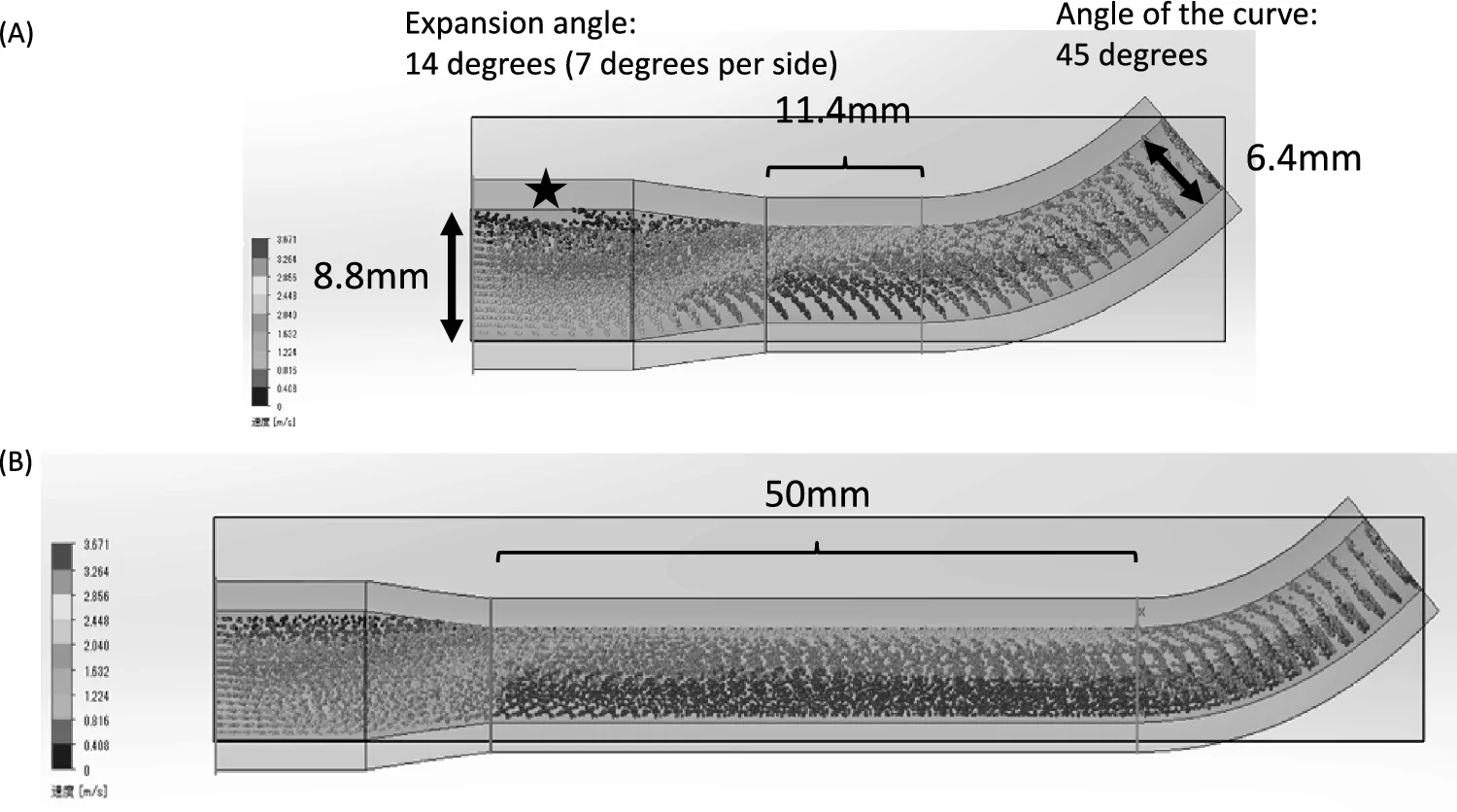
The flow of the circuit before the enlarged flow path. **A** The flow when the circuit before the enlarged flow path is bent. Large-scale flow separation and reverse flow are observed in the enlarged flow path (★). **B** The flow when the circuit before the enlarged flow path is not bent.

ECMO circuits used in children are typically constructed with 1/4-inch tubing. However, the inlet and outlet ports of centrifugal pumps available in Japan are 3/8-inch, necessitating flow path enlargement near these ports. Furthermore, depending on the relative positions of the bed and console, the circuit may become sharply kinked immediately before the centrifugal pump, making the enlarged flow path prone to promoting thrombus formation. Kurosawa et al. performed CFD analysis to investigate methods for preventing this thrombus formation. They reported that a smaller expansion angle at the enlarged flow path section can suppress large-scale flow separation and velocity reduction, thereby inhibiting thrombus formation. They also noted that bending the circuit before the enlarged flow path causes large-scale flow separation and reverse flow at the enlarged flow path, making thrombus formation more likely. Conversely, when the circuit before the enlarged flow path is not bent, no large-scale flow separation or reverse flow is observed (Fig. 1). Therefore, to prevent thrombus formation, it is crucial to configure the circuit to avoid kinking near the enlarged flow path [13]. Thus, it is necessary to understand that controlling anticoagulant dosage only is insufficient to prevent thrombus formation in the ECMO circuit; attention must also be paid to step-ups at connector junctions and circuit configuration.

### Flow rate

Mayer et al. reported that when perfusing heparinized blood in vitro at 0.3, 0.5, and 0.7 L/min, increased tissue factor on leukocytes and extracellular vesicles was observed at 0.3 and 0.7 L/min, and these were associated with thrombus formation [14]. It is important to note that a higher flow rate does not necessarily prevent thrombus formation.

## Hemolysis

When hemolysis occurs, free hemoglobin increases in plasma. Hemoglobin taken up by tubular epithelial cells is broken down into heme and globin. Heme exhibits toxicity to tubular epithelial cells, causing acute kidney injuly. Furthermore, it has been suggested that hemolysis depletes nitric oxide (NO), adenosine, and ADAMTS13, leading to peripheral vasoconstriction and platelet thrombus formation, which can cause multi-organ dysfunction beyond the kidneys [15]. Although some reports indicate hemolysis is not an independent factor affecting survival [3], it remains a complication requiring careful attention during ECMO [16, 17].

In a 2012 study by Barrett et al. using propensity score matching, comparing 88 infants undergoing V-A ECMO with centrifugal pump versus 88 infants with roller pump, the odds ratios (OR) for hemolysis, hyperbilirubinemia, hypertension, and acute kidney injury in infants using centrifugal pumps were 7.7 [2.8–21.2], 20.8 [2.7–160.4], 3.2 [1.3–8.0], and 2.4 [1.1–5.6], respectively. This suggests that the use of centrifugal pumps in pediatric patients may contribute to increased rates of hemolysis and multi-organ dysfunction [18]. The centrifugal pumps used in this study were the Biomedicus BP-80, Biomedicus BP-50, Jostra Rotaflow, and Levitronix CentriMag. The analysis limited to Rotaflow and CentriMag showed higher ORs for hemolysis, hyperbilirubinemia, and acute kidney injury, but these were lower than in the analysis including BP-80 and BP-50. This suggests that the higher incidence of hemolysis and organ dysfunction may be influenced by the bearings of the centrifugal pumps. Reports on extracorporeal circulation using MiECC (Minimum invasive Extra-Corporeal Circulation) in adult cases also indicate that hemolysis is lower with magnetic levitated centrifugal pump than with double-pivot type centrifugal pump [19], suggesting that bearing structure does have some influence. However, as mentioned above, even in the analyses limited to Rotaflow and CentriMag, the ORs for hemolysis, hyperbilirubinemia, and acute kidney injury remained high. Furthermore, at our institution, which uses a mono-pivot type centrifugal pump, the incidence of hemolysis in pediatric and neonatal cases is higher compared to adult cases. Therefore, the influence of the bearing on hemolysis appears to be limited.

**Fig. 2:**
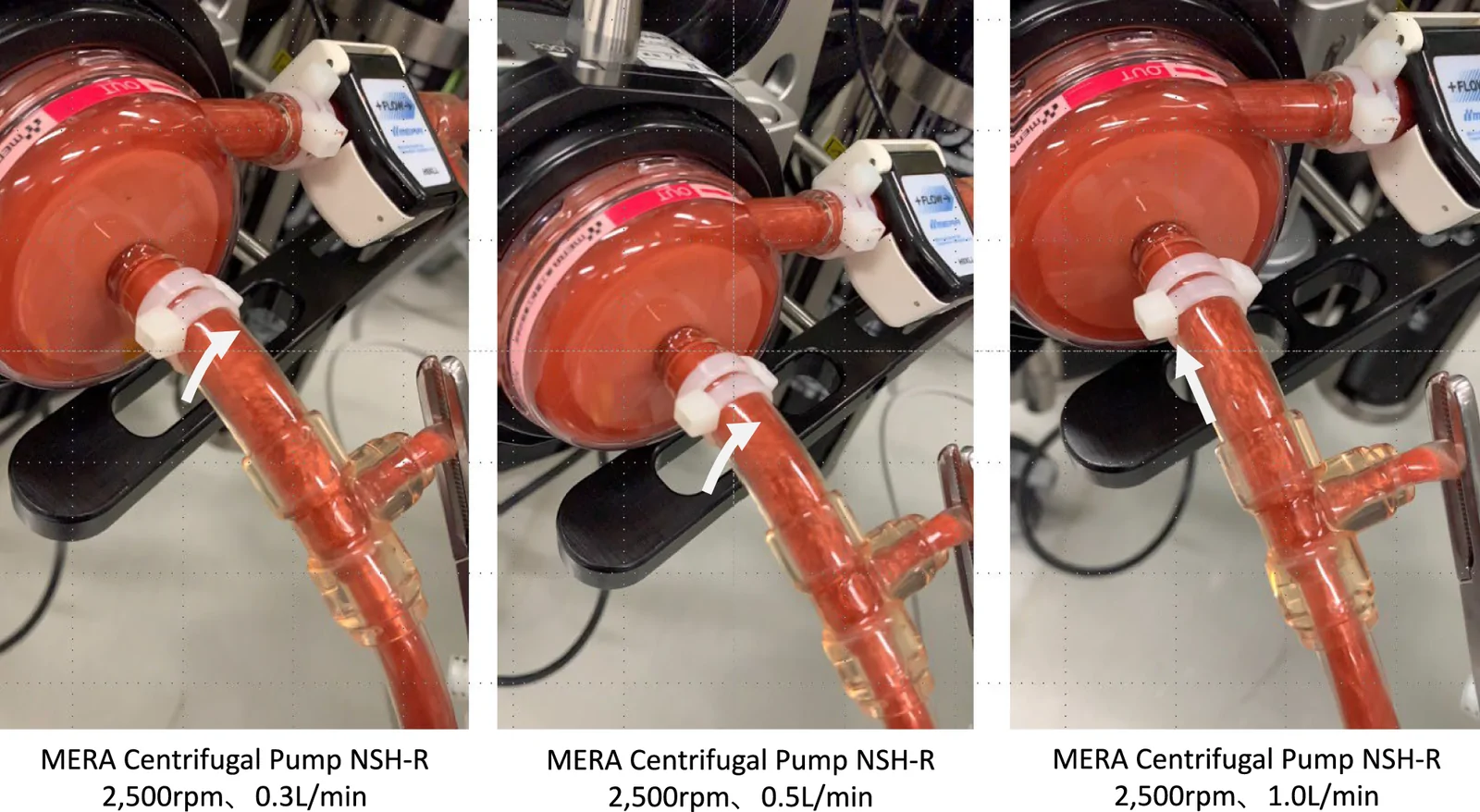
Flow pattern of fluid at the inlet side of a centrifugal pump when circulating water containing dispersed mica particles. Up to approximately 0.5 L/min, a swirling flow pattern in the direction of the arrow was observed. However, at flow rates of 0.8 L/min or higher, the fluid was confirmed to flow smoothly into the centrifugal pump.

Schöps et al. performed in-silico analysis at the same centrifugal pump speed and demonstrated that the flow rate of 1 L/min resulted in a higher recirculation rate and increased hemolysis indicators compared to the flow rate of 4 L/min. They also reported that while no flow exceeding 50 Pa shear stress was detected in the centrifugal pump at 4 L/min, the flow exceeding 150 Pa—the threshold for hemolysis—was confirmed at 1 L/min [20]. The results of the hydrodynamically levitated centrifugal pump developed by Department of Artificial Organs at the National cerebral Cardiovascular Center Research Institute (Osaka, Japan) also showed that at the same rotational speed, a decrease in flow rate increases the proportion of flow passing through the gap between the housing and impeller. This indicates that the recirculation rate in the centrifugal pump is higher at lower flow rates [21]. Furthermore, observing the flow at the inlet side of the centrifugal pump while circulating water containing dispersed mica particles revealed that up to approximately 0.5 L/min, the fluid did not enter the centrifugal pump smoothly, and a swirling flow pattern was observed. However, at 0.8 L/min or higher, the fluid flowed smoothly into the centrifugal pump (Fig. 2). Based on the above, the primary cause of increased hemolysis incidence when using centrifugal pumps at low flow rates is considered to be the high recirculation rate and high shear stress on the blood. Therefore, while using roller pumps may be more optimal for pediatric patients, modifying existing ECMO systems is not straightforward. Thus, when hemolysis occurs during ECMO using a centrifugal pump in pediatric patients, the only management option is prompt administration of haptoglobin preparations to supplement circulating haptoglobin levels as a symptomatic treatment. In our institution, we measure plasma free hemoglobin, and if the levels are 0.05 g/dL or higher, we consider administering haptoglobin. Because urine color change or elevation of urinary free hemoglobin levels follows the elevation of plasma free hemoglobin levels [22] or urine color change is not detected at all, measuring plasma free hemoglobin is useful. Furthermore, it is known that free hemoglobin irreversibly binds to NO, depleting arginase—an enzyme essential for NO synthesis in the vascular endothelium—leading to a physiological NO deficiency. In contrast, exogenous NO administration oxidizes hemoglobin heme iron to methemoglobin (MetHb). Since oxidized hemoglobin does not bind NO, this helps maintain circulating NO levels, potentially reducing the risk of organ dysfunction [23]. Therefore, inhaled NO therapy may be considered as a strategy to reduce the incidence of complications during hemolysis.

The optimal solution to reduce the incidence of hemolysis is to use centrifugal pumps designed for low flow ranges. However, given the current situation where such centrifugal pumps are not manufactured in Japan, it seems necessary to proceed with appropriate management, bearing in mind that using centrifugal pumps in pediatric patients increases the susceptibility to hemolysis.

## Future challenges

We have outlined considerations for pediatric ECMO management from the perspectives of thrombosis prevention and hemolysis risk. Pediatric cases not only present challenges in anticoagulation management, but the use of centrifugal pumps at low flow rates likely exacerbates both thrombosis and hemolysis. Consequently, the use of centrifugal pumps itself appears to further complicate ECMO management in children. Although the relatively small number of pediatric cases compared to adults hinders the development of an optimal pediatric ECMO system, reducing complications and improving outcomes necessitates future development of a pediatric ECMO system, including centrifugal pumps optimized for low flow range.
